# Impact of carbamazepine on *SMARCA4* (BRG1) expression in colorectal cancer: modulation by *KRAS* mutation status

**DOI:** 10.1007/s10637-024-01418-2

**Published:** 2024-03-06

**Authors:** Aaron Shaykevich, Danbee Chae, Isaac Silverman, Jeremy Bassali, Netanel Louloueian, Alexander Siegman, Gargi Bandyopadhyaya, Sanjay Goel, Radhashree Maitra

**Affiliations:** 1 Department of Biology, Yeshiva University, New York, NY 10033 USA; 2 Rutgers Cancer Institute of New Jersey, New Brunswick, NJ USA

**Keywords:** BRG1, *SMARCA4*, *KRAS*, Carbamazepine, Cancer, SWI/SNF, ULK1

## Abstract

*SMARCA4* is a gene traditionally considered a tumor suppressor. Recent research has however found that *SMARCA4* likely promotes cancer growth and is a good target for cancer treatment. The drug carbamazepine, an autophagy inducer, was used on colorectal cancer cell lines, HCT1116 and Hke3 (*KRAS* mutant and wildtype). Our study finds that Carbamazepine affects *SMARCA4* levels and that this effect is different depending on the *KRAS* mutation status. This study analyzes the effect of carbamazepine on early-stage autophagy via ULK1 as well as simulates the docking of carbamazepine on *KRAS,* depending on the mutation status. Our study highlights the therapeutic uses of carbamazepine on cancer, and we propose that carbamazepine in conjunction with other chemotherapies may prove useful in targeting *KRAS-*mutated colorectal cancer.

**Supplementary Information:**

The online version contains supplementary material available at 10.1007/s10637-024-01418-2.

## Introduction

Colorectal cancer (CRC) is the third most common and second most deadly cancer in the United States among both men and women [1]. Among those diagnosed with CRC, approximately 40% have a mutation to the *KRAS* gene, encoding the K-ras protein [2]. K-ras is a GTPase involved in signal transduction and a key component of the Ras/Raf pathway. K-ras also plays an activating role as part of the Ras family in the ERK1/2 and PI3K signaling pathways, both of which have been linked to tumorigenesis when overexpressed [3]. Active mutations to *KRAS* have also resulted in suppression of apoptosis suppression and cell proliferation [4]. Overall, the presence of a mutated *KRAS* gene often leads to worse prognoses in CRC patients [2]. Few treatments are available for patients with the *KRAS* mutation, and only two *KRAS-*mutant-inhibitors, Sotorasib and Adagrasib, are currently FDA approved, both of which specifically target the G12C mutation [5–7]. While several clinical trials are currently in progress for other *KRAS-*inhibitors, there is still the prevalent issue of acquired resistance to them [5–7]. Therefore, additional methods of targeting *KRAS* mutated CRC are necessary for effective treatment.

*SMARCA4* (encoding the protein BRG1) is a catalytic subunit of the SWI/SNF chromatin remodeling complex [8]. Interestingly, the SWI/SNF complex mechanism has been proven to play a role in transcription through nucleosome remodeling [9]. In relation to cancer, *SMARCA4* has been thought to act as a tumor suppressor. However, recent research highlights that *SMARCA4* assists in tumor proliferation [10]. In many cancers *SMARCA4* is found to be mutated and likely acts as an oncogene [11–13]. Notably in CRC, it has been observed that an inhibition of BRG1 reduced the proliferation of tumorigenesis [14]. As a result of such discoveries indicating beneficial outcomes of BRG1 inhibition, recent research has now focused on the effects of both BRG1 and *SMARCA4*-inhibition, as well as viable methods of doing so clinically. Significant correlations have already been made between the upregulation of *SMARCA4* and *KRAS* mutations in small-cell lung cancer. Often *SMARCA4* was found to be co-mutated with the G12C *KRAS* mutation in patients and the G12D *KRAS* mutation in mice. However, targeting *SMARCA4*, regardless of *KRAS* mutation, may prove a successful method of treating cancers. The knockout of the *SMARCA4* gene has been shown to induce apoptosis and kill cancer cells [10, 15, 16]. However, there are few drugs that can inhibit *SMARCA4* expression, and no drugs that target *SMARCA4* exclusively in cancer. Therefore, novel methods of *SMARCA4* inhibition may benefit CRC patients harboring a *KRAS* mutation.

One drug that has the potential to affect cancer growth is carbamazepine (CBZ). CBZ is a drug primarily used in patients with epilepsy [17]. However, research has found the CBZ has a strong impact on significant cellular pathways such as autophagy [11–13], a process promoting cell survival by recycling damaged cell components. This suggests that CBZ may have other uses in cancer treatment, in particular it may help against *KRAS-*mutated CRC by working against *KRAS*. By analyzing the effect and interaction of CBZ on genes involved in cancer proliferation, such as *SMARCA4*, a better understanding can be established on the tumor suppressing or promoting effect CBZ has on CRC.

## Methods and materials

### Cell lines

Two CRC cell lines, HCT116 and Hke3, were used in this study. HCT116 is a *KRAS* mutant cell line, harboring a G13D mutation, while Hke3 is *KRAS* WT. HCT116 and Hke3 are isogenic, and Hke3 was originally made by reverting the *KRAS* mutation in HCT116 [18]. HCT116 were purchased from the American Type Culture Collection (ATCC®). Hke3 cell lines were obtained from Dr. Takehiko Sasazuki (Medical Institute of Bioregulation, Kyushu University).

### Cell culture

Cell lines were cultured in Roswell Park Memorial Institute (RPMI) 1640 media (Gibco™, Catalog #: 11875093), with 10% Fetal Bovine Serum (GemCell™, Catalog #: 100–500), 1% Non-Essential Amino Acids (Gibco™, Catalog #: 11140050), 2% HEPES buffer (Gibco™, Catalog #: 15630080), 1% Antibiotic–Antimycotic (Gibco™, Catalog #: 15240062), and 0.4% gentamicin (Gibco™, Catalog #: 15710064). The cells were maintained in an atmosphere of 5% CO2 at 37 °C and passaged according to ATCC®’s recommended protocol.

### Carbamazepine (CBZ) preparation

Carbamazepine powder was purchased from (Supelco™, Catalog #: PHR1067-1G). The CBZ powder was dissolved in absolute methanol at a concentration of approximately 2 mg/ml (8.5mMol) and placed into single-use aliquots at -20 °C. At the time of treatment one aliquot of the Carbamazepine solution was diluted with media to 500uM.

### Cell treatment

Cells were cultured until 70% confluency, trypsinized (Corning™, Catalog #: 25–053-CI), and spun down into cell pellets. Cells were then counted using the Countess™ II Automated Cell Counter (Invitrogen™, Catalog #: AMQAX1000) with Trypan Blue solution (Sigma-Aldrich™, Catalog #: T8154) as per the manufacturer’s protocol. Four plates of 5–7 million cells were made in a 100 mm plate (Denville™), two HCT116 and two Hke3, and allowed to remain for 24 h in 9 mL of cell culture media. After that time, one plate of each cell type was then treated with 1 mL of the 500uM Carbamazepine solution (final concentration in media = 50uM). 6 or 24 h after treatment, both the untreated and treated cell lines were trypsinized and harvested. 25% of the pellet was set aside for RNA extraction, while 75% was set aside for protein extraction.

### RNA extraction and quantification

RNA was extracted from the cell pellets using the Invitrogen™ PureLink™ RNA Mini Kit (Invitrogen™, Carlsbad, CA, USA, Catalog #: 12183018A) as per the manufacturer’s protocol. The purified RNA was then placed into single-use aliquots and stored at − 80 °C. The concentration of the extracted RNA was quantified using a Thermo Scientific™ NanoDrop 1000 (Thermo Scientific™, Catalog #: 2353–30-0010). The 260/280 of the RNA was checked and the RNA was only kept if the range of 260/280 was between 1.9 and 2.1.

### cDNA synthesis

A total of 1.5 μg of the extracted and quantified RNA was synthesized into cDNA using the iScript Reverse Transcription Supermix for RT-qPCR (Bio-Rad™, Catalog #: 1708841) as per the manufacturer’s protocol. A T100™ Thermal Cycler (Bio-Rad™, Catalog #: 1861096) was used to run the reaction. 100 μL of DEPC-treated water (Thermo Scientific™, Catalog #: R0601) was then added to each sample. The synthesized cDNA was then estimated using the Thermo Scientific™ NanoDrop 1000 and diluted to 25 ng/ul with DEPC-treated water and placed into single-use aliquots and stored at − 80 °C.

### Quantitative polymerase chain reaction (qPCR)

Primers were purchased from Sigma-Aldrich™ (Easy Oligo) and arrived pre-diluted in deionized water at a concentration of 100 µM. The primers were made into single-use aliquots upon arrival and stored at − 20 °C. The sequences of the primers used can be seen in Table 1.

**Table 1:** Primer Sequences

Primer Name	Forward	Reverse
SMARCA4	TACAAGGACAGCAGCATGG	TAGTACTCGGGCAGCTCCTT
ULK1	CACGCCACATAACAGACAAAAATAC	ACAAGGTGAGAATAAAGCCATCAAG
GAPDH	CTTTTGCGTCGCCAG	TTGATGGCAACAATATCC

Primers were prepared for qPCR by adding 10 µL of forward primer, 10 µL of reverse primer, and 180 µL of Thermo Scientific TM DEPC treated water (Thermo Scientific, Catalog #: FERR0601) (5 µM final concentration of forward and reverse primer). A total of 1 µL of the prepared 5 µM primer mix, 4 µL of the synthesized cDNA, and 5 µL of the Applied Biosystems™ PowerUp™ SYBR™ Green Master Mix (Applied Biosystems™, Catalog #: A25918), were added to each well of a qPCR tube set (Bio Molecular Systems™, Catalog #: 71–107) (final reaction mix contained a 500 nM concentration of each primer and 100 ng of cDNA). Quantabio™ Q cycler was used to run the qPCR (Quantabio™, Catalog #: 95900-4C). All reactions were prepared in triplicates.

Data analysis was performed by the ΔΔCT method. ΔCT was first calculated by subtracting each sample’s GAPDH CT value from its average target gene CT value. ΔΔCT was calculated by subtracting ΔCT of the untreated cell from the ΔCT of the treated cell. The ΔΔCT values were then converted to fold values using 2^(-ΔΔCT).

### Protein extraction

Cell Extraction Buffer (Invitrogen™, Catalog #: FNN0011) and 10 µL of Thermo Scientific™ Halt™ Protease and Phosphatase Inhibitor Cocktail EDTA-free (100X) (Thermo Scientific™, Catalog #: 78445) was added to a microcentrifuge tube per 1 mL of cell Extraction Buffer. Each cell pellet was suspended in 250 µL of the freeze–thaw lysis buffer with protease and phosphatase inhibitor. The cell pellets were then dipped in liquid nitrogen for 10 s, allowed to thaw, and then vortexed. This was repeated three times. After the third freezing with liquid nitrogen, cells were placed on ice to thaw. The cell pellets were then placed in a microcentrifuge at max speed for 45 min at 4 °C. The supernatants were then placed into single-use aliquots at − 80 °C.

### Protein estimation

Protein was estimated using a Bradford assay by combining in each 1.5 mL tube: 500uL of H_2_O, 495uL of Bradford, and 5uL of protein sample or BSA standard (or an additional 5uL Bradford for the plate blank). 600uL of this mixture was then plated in a 96 well plate (Corning™, Catalog #: 3598) at 200uL per well. The plate was then read using a SpectraMax Mini Multi-mode Microplate reader (Molecular Devices™, Catalog #: 76640–506) for absorbance at 595 nm. All values subtracted the plate blank, and the triplicate values were averaged together. A standard curve was made using BSA standards and protein was estimated using this curve.

### Western blot

40ug of protein from each sample was used. Protein was prepped by mixing 1 part protein and 1 part 2 × laemilli sample buffer (Bio-Rad™, Catalog #: 1610737) and placed in boiling water for 10 min. Each sample was loaded in a 4–20% gel (Bio-Rad™, Catalog #: 4561094) and 2 uL of Magic marker (Invitrogen™, Catalog #: LC5602) and 10uL of protein ladder (Thermo Scientific™, Catalog #: 26616) was loaded as well. Transfer was done using a wet transfer at 80 mV for 1 h to a nitrocellulose membrane (Bio-Rad™, Catalog #: 1620215). Membranes were blocked in 1% BSA in TBS for an hour and then then allowed to sit in antibody overnight. The primary antibody for BRG1 was Invitrogen™ BRG1 Monoclonal Antibody (GT2712) at a 1:1000 dilution (Invitrogen™, Catalog #: MA5-31550). The primary antibody for ULK1 was Invitrogen™ ULK1 Recombinant Rabbit Monoclonal Antibody (JA58-36) at a 1:1000 dilution (Invitrogen™, Catalog #: MA5-32699). The primary antibody for Beta actin was Abnova ACTB monoclonal antibody (M01) clone 3G4-F9 at a 1:750 dilution (Abnova, Catalog #: H00000060-M01). The antibody detection was done using the Pierce™ Fast Western Blot Kit (Thermo Scientific™, Catalog #: 35050) and followed the manufacturer’s protocol. The ChemiDoc MP imaging system (Bio Rad™, Catalog #: 1708280) was used to detect chemiluminescence.

### TCGA gene correlation analysis

The GEPIA online website was used to analyze the correlation of genes in CRC patients from The Cancer Genome Atlas (TCGA) database [19]. We assessed the correlation between gene expression of *SMARCA4* and *KRAS*. This was done on the GEPIA website by selecting the Multiple Gene Analysis tab, selecting Correlation Analysis, and then selecting the following options: Gene A = *SMARCA4*, Gene B = *KRAS,* Normalized by gene = TUBA1A, Correlation Coefficient = Spearman, and Used Expression Datasets = COAD Tumor & READ Tumor, and then separately selected Used Expression Datasets = COAD Normal & READ Normal.

### GDC gene expression and Kaplan Meier plots

The Xena online website was used to analyze the expression of genes in CRC patients from TCGA database [20]. This was done by selecting the “GDC Pan-Cancer” dataset, limiting the “cancer type” to COAD or READ, and keeping only “primary tumor” or “solid tissue normal.” Three categories were created as follows: **Solid Tissue Normal**: Data with “sample_type” labeled as Solid Tissue Control. ***KRAS****-***wt CRC**: Data with “sample_type” labeled as primary tumor and no mutation to the *KRAS* gene. ***KRAS****-***mut CRC**: Data with “sample_type” labeled as primary tumor and one of the following mutations to the *KRAS* gene: G12A, G12C, G12D, G12R, G12S, G12V, G13C, G13D. Any sample without gene expression reported was removed. Cancer samples with “Null” for *KRAS-*mutation status were removed. The final sample size was n = 539 consisting of 51 solid tissue, 309 *KRAS-wt* CRC, and 179 *KRAS-*mut CRC.

We selected the three dots above the box containing the 3 subgroups of samples and selected differential expression. For our first calculation of mRNA changes in cancer we set subgroup A to include both cancer groups and subgroup B to include the Solid Tissue control. Our next calculation, of mRNA change in the presence of a *KRAS* mutation, we set subgroup A to include the *KRAS-*mut data and subgroup B to include the *KRAS-*wt data. The advanced settings were not changed. A file including Log(2)FC, p-value, and adjp is given, and fold change of the target gene is then calculated. In order to visualize these changes, the mRNA expression of the target genes were opened. The “view as chart” symbol was selected and “compare subgroups” was then selected. “Show data from” included the target gene and “subgroup samples by” included our three groups of samples.

For the Kaplan Meier plot, three separate tabs were made, each one including only one of the subgroups. *SMARCA4* mRNA expression was then opened on each tab, the three dots above the box were selected, and “Kaplan Meier Plot” was selected. The data set into quarterlies, and the cutoff was selected to 1500 days. The p-value is calculated by Xena using a log-rank test.

### Molecular dynamic simulations and structural and energetic analysis

The AI AlphaFold predicted structures for human *KRAS* (ID: AF-P01116-F1) and human *SMARCA4* (ID: AF-P51532-F1) were downloaded from the Uniprot database in Protein Data Bank (PDB) format [21, 22]., Each file was uploaded separately to PyMol to visualize the 3D structures. Each protein file contained a single protein chain (Chain A). *KRAS* contained 189 residues and *SMARCA4* contained 1647 residues. Chain A for both *SMARCA4* was renamed “Chain B” to act as a “ligand” in a complex with Chain A of *KRAS,* serving as a “receptor.” To create the G13D mutation in *KRAS,* in PyMOL the Gly13 residue was mutated to Asp13 using protein mutagenesis. This was saved as a separate pdb file.

Wildtype *KRAS* and G13D were each docked to each other using the ClusPro server [23–26]. Balanced structure “0” was downloaded as the complex structure for each docking. The proteins were again separated into separate chain files using PyMOL.

Using GROMACS, a topology file was created for *KRAS-*wt /G13D and *SMARCA4*. An OPLS-AA/L all atom force field was used. The *SMARCA4* topology file was combined with the *KRAS-*wt topology to build the complex. A cubic box was generated and solvated. Ions were added to neutralize any charge in the complex if present. An energy minimization was run to reduce unfavorable sterics in the complex structure. NVT and NPT equilibrations were then used to respectively stabilize the temperature and pressure of the environment before running a 10 ns molecular dynamics simulation. Following the simulation, a trjconv command was used to correct any jumps of the protein around the box.

Root mean squared deviation (RMSD) was calculated for the carbon backbone to determine how much the complex moved from its original position, showing overall stability. Root mean square fluctuation (RMSF) was calculated for each protein in the complex in each simulation to determine the average displacement of their residues. Radius of gyration (Rg) was calculated for each protein in the complex as a measure of each of their overall structural compactness throughout the simulation while interacting.

gmx_MMPBSA software was used to calculate a per-residue decomposition analysis [27]. A MMGBSA (Generalized Born model) was used to produce energy values for the complex, the receptor *KRAS* /G13D), the ligand (*SMARCA4*), and the delta energy. The complex energy represented the bound state energy, and the receptor and ligand energies represented the unbound energies for *KRAS* /G13D and *SMARCA4*. The delta energy represented the overall binding strength. Delta energy *(interaction energy)* = Total Complex *(bound state)*—[Receptor + Ligand] *(unbound states)*. Simulation energy values were represented in tables and graphs generated by the software.

### Protein-drug docking

*In-silico* protein-drug dockings were accomplished using the CB-Dock2 server [28, 29]. The *KRAS-*wt, G13D, and *SMARCA4* pdb files generated for the simulations were reused. The model structure for carbamazepine (ID: N6W) was downloaded from the RCSB database. The model structures for *trans*-carbamazepine diol and carbamazepine-o-quinone were created using the CB-Dock2 server ligand drawing function. *KRAS*-wt*,* G13D, and *SMARCA4* were each uploaded to the server with each form of carbamazepine for cavity detection and docking. The CB-Dock2 server generated five possible binding conformations for each protein-drug pair, ranked by their energetic favorability. The highest ranked conformation was chosen for analysis.

### Statistical analysis

Statistical analysis of western blot and qPCR data was performed using Microsoft™ Office Excel. For fold change, a two-tailed one-sample t-test was used. When comparing fold changes, a two-tailed two-sample t-test was used. Outliers were determined and removed using Iglewicz and Hoaglin’s outlier test with modified z-scores using the outlier criterion of a modified z-score ≥ 3.5.

## Results

### Depending on the presence of a KRAS mutation, the expression of SMARCA4 increases in CRC and affects overall survival. SMARCA4 altered interactions with KRAS wildtype vs KRAS mutant in sillico

**Fig. 1:**
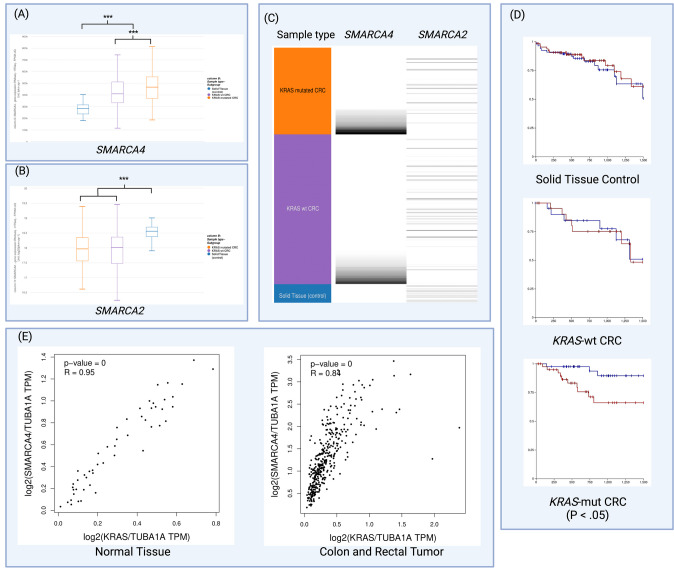
**A** *SMARCA4* mRNA expression is increased in CRC and further increased in *KRAS-*mutated CRC in patient datasets. **B** *SMARCA2* mRNA expression decreased in CRC in patient datasets. **C** A visual representation of the mRNA expression of *SMARCA4* and *SMARCA2* in differing tissue types. Darker lines represent higher expression. **D** A Kaplan Meier plot of overall survival for the first 1500 days in different tissue types. Blue: low *SMARCA4*, Red: High *SMARCA4*. **E** Positive correlation between *KRAS* and *SMARCA4* in normal tissue and colon or rectal tumors (p = 0)

In order to better understand the method by which *KRAS* mutated CRC promotes cell survival, the TCGA COADREAD clinical patient dataset was analyzed with assistance from the UCSC Xena software. We first analyzed the difference in gene expression of normal tissue vs colorectal cancer data. We then focused on comparing the gene expression of *KRAS-*mut CRC vs *KRAS-wt* CRC. We found that *SMARCA4* was indeed overexpressed in cancer patients by 59% (p = 1.81e-16, adj p = 1.71e-15). When comparing *SMARCA4* expression in *KRAS-*mut vs *KRAS-wt* it was found that *KRAS-*mutant CRC has a 15% increase in *SMARCA4* expression (p = 5.03e-5, adj p = 0.002) (Fig. 1A). We found that the homolog *SMARCA2* decreased expression in cancer patients by 36% (p = 3.87e-10, adj p = 2.05e-09) (Fig. 1B). The gene expression can be expressed visually using Xena (Fig. 1C). This confirms previous literature that *SMARCA4* uniquely acts as a tumor promoter in colorectal cancer [10, 16], and may present a new role of *SMARCA4* in tumorigenesis in CRC with a *KRAS* mutation.

A plot of overall survival in *KRAS-*mutant CRC shows that high *SMARCA4* expression significantly impacts survival probability in the first 1500 days compared to low *SMARCA4* expression (p = 0.015). However, similar to the control sample, high *SMARCA4* levels in *KRAS-wt* CRC do not significantly impact survival (Fig. 1D). This demonstrates a connection between *KRAS-*mutation and *SMARCA4*’s activity. Using GEPIA, we were able to analyze the correlation between *KRAS* and *SMARCA4* in both COADREAD as well as normal tissue. In both the control as well as in cancer patients it was found a positive correlation between *KRAS* and *SMARCA4* (p = 0, R = 0.95 and p = 0, R = 0.84 respectively) (Fig. 1E).

Within the complexity of the cell, there is potential for *KRAS* interaction with *SMARCA4*. If the two proteins interacted, an MDS details the structural and energetic components of the interaction. A comparison of *KRAS-*wt and G13D interactions with *SMARCA4* demonstrates the effect of the mutation. The RMSD of each complex indicated that both systems were well-equilibrated (Supplementary Fig. S1A). Interestingly, the G13D complex initially had a higher deviation than the *KRAS-*wt complex from 0.5 ns—3 ns. It again had slightly higher deviation from 4 ns—5 ns. Between 6 ns—7 ns the *KRAS-*wt complex actually had a slightly higher deviation. The *KRAS-*wt complex ultimately equilibrated at 2.12 nm, which was higher than the equilibrium of the G13D complex at 1.97 nm. A higher RMSD value indicates lesser stability at those points in the simulation. The RMSF of *SMARCA4* with each *KRAS*-wt and G13D are compared (Supplementary Fig. S1B). *SMARCA4* also had slightly more fluctuation when interacting with *KRAS-*wt, except around residues 300–400, where there was significantly more fluctuation with *KRAS-*wt. In the Rg of both complexes *SMARCA4* increased in compactness throughout the simulation, indicated by their negative slopes. However, *SMARCA4* with *KRAS-*wt had lower Rg values than *SMARCA4* with G13D, signifying the former had higher compactness. This trend was also observed for *KRAS-*wt and G13D themselves in which the former had lower Rg values throughout the simulation (Supplementary Fig. S1C). Energetically, both simulations produced stable binding patterns with large negative values. Line plots of the total complex energies throughout the simulations indicate that the *KRAS-*wt complex has less total energy than the G13D complex (Supplementary Fig. S1D). The *KRAS-*wt complex has a gradual decrease in energy, signifying an increase in favorable energy with a large standard deviation. However, the G13D complex remains more consistent with less of a standard deviation (Supplementary Table S1). Line plots of the delta energies indicate the overall strength of the binding energy in each simulation. The *KRAS-*wt complex has a gradual increase in favorable energy from ~ -80 kcal/mol to ~ -200 kcal/mol over the course of the simulation with an average energy of -133.5 kcal/mol. The G13D complex has a stronger binding energy of -150.6 kcal/mol and a much greater fluctuation in binding strength throughout the simulation (Supplementary Fig. S1E). The amount of individual residues involved in the delta energy reduced from 95 in the *KRAS-*wt complex to 84 in the G13D Complex.

### The expression of the protein encoded by SMARCA4, BRG1, is lowered in KRAS-mut cancers treated with CBZ and upregulated in KRAS-wt depending on the time after treatment

**Fig. 2:**
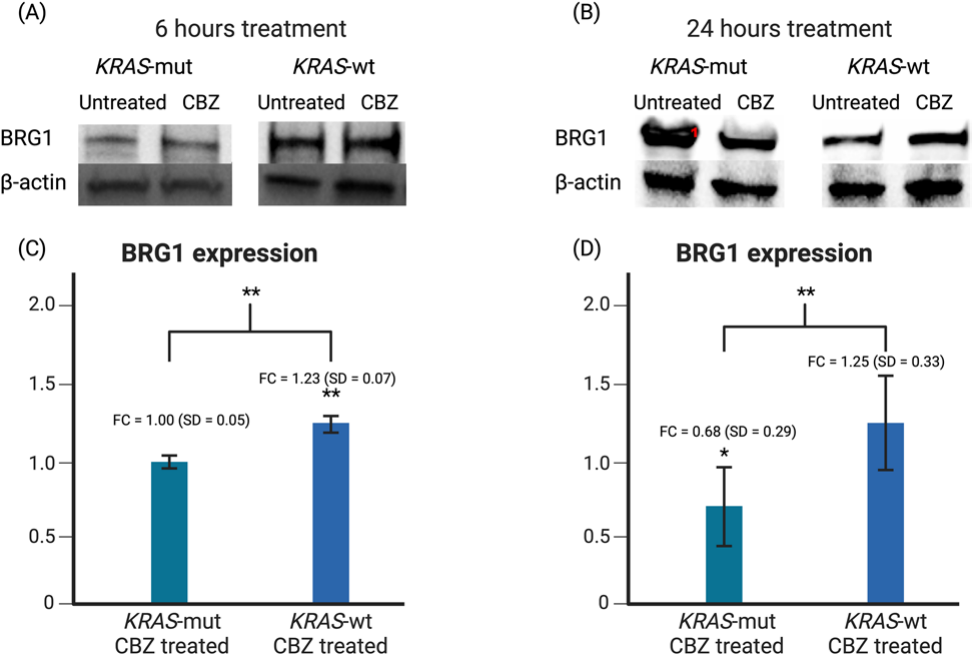
**A** After 6 h, BRG1 expression is shown to be upregulated in *KRAS-*wt CRC and not significantly affected in *KRAS-*mut CRC. **B** After 24 h, BRG1 expression is shown to be downregulated in *KRAS-*mut CRC and not significantly affected in *KRAS-*wt CRC. **C** Densitometric analysis of 5 western blot experiments were averaged together and the mean and standard deviation were calculated. In *KRAS-*wt CRC, BRG1 expression has a fold change of 1.23 which is statistically significant to untreated CRC (p < 0.01) and significant to *KRAS-*mut CRC (p < 0.01). **D** Densitometric analysis of 6 western blot experiments were averaged together and the mean and standard deviation were calculated. In *KRAS-*mut CRC, BRG1 expression has a fold change of 0.68 which is statistically significant to untreated CRC (p < 0.05) and significant to *KRAS-*wt CRC (p < 0.01)

In order to understand the possible effect of CBZ on *SMARCA4* expression in *KRAS-*mut and *KRAS-wt* tumors, CRC cell lines were treated with 50uM of CBZ for 6 or 24 h. The fold change between the untreated and treated showed that in *KRAS-*mut cells, BRG1 expression lowered at 24 h (p < 0.05). In *KRAS-wt*, BRG1 expression was significantly raised at 6 h (p < 0.01) but not at 24. The fold change between *KRAS-*mut and *KRAS-wt* was statistically significant at both 6 and 24 h (p < 0.01 for both). This demonstrates the opposite effect of CBZ treatment on *KRAS-*mut and *KRAS-wt* CRC BRG1 expression (Fig. 2).

### SMARCA4 mRNA levels is lowered in KRAS-mut cancers treated with CBZ and raised in KRAS-wt

**Fig. 3:**
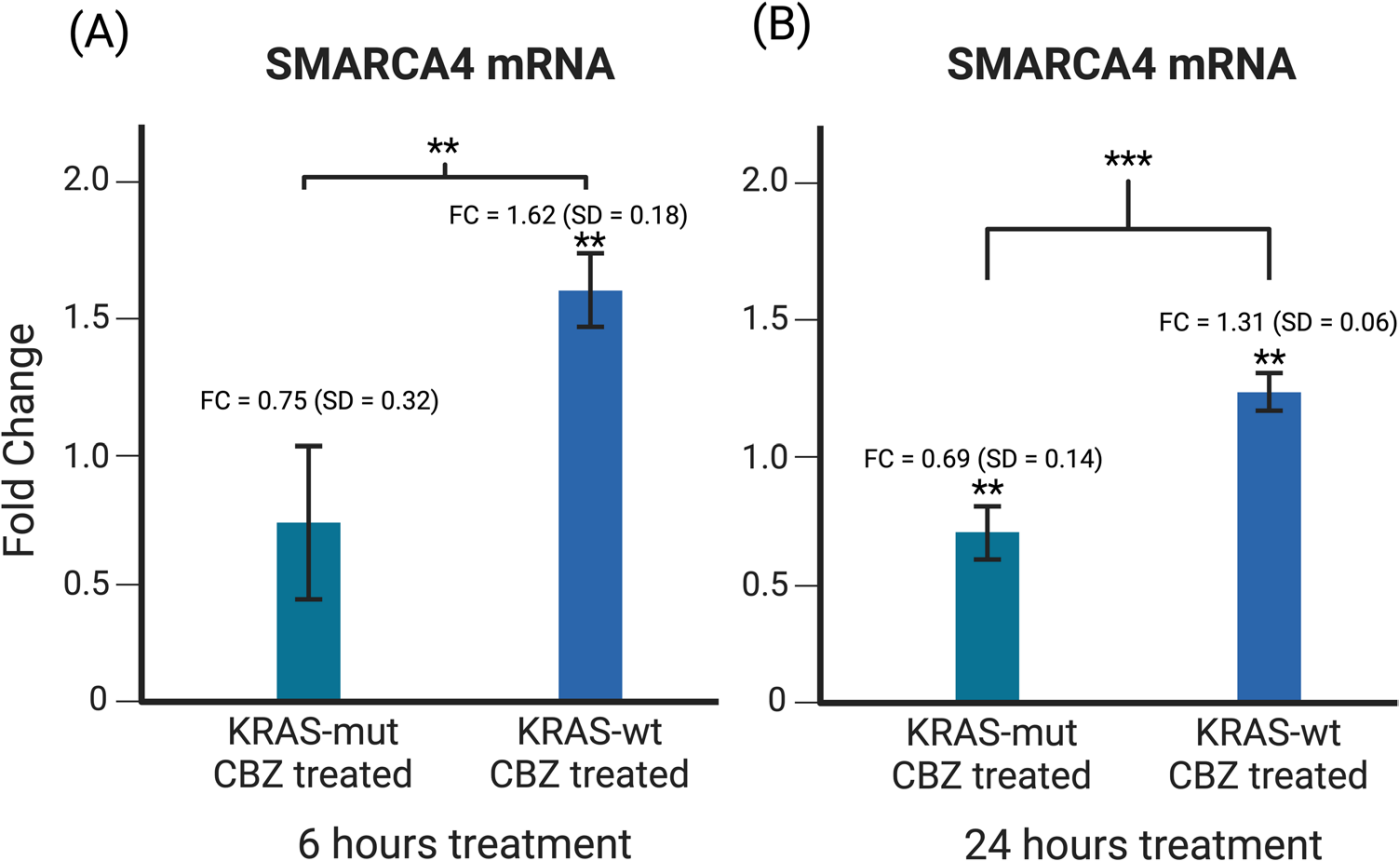
**A** Results are an average of 4 separate experiments. After 6 h of treatment, *SMARCA4* mRNA levels increased significantly in *KRAS-*wt cells treated with CBZ (p < 0.01). These levels are significantly higher than in *KRAS-*mut cells treated with CBZ (p < 0.01). **B** Results are an average of 5 separate experiments. After 24 h of treatment, *SMARCA4* mRNA levels increased significantly in *KRAS-*wt cells treated with CBZ (p < 0.01) and decreased significantly in *KRAS-*mut cells treated with CBZ (p < 0.01). The difference between *KRAS-*mut cells and *KRAS-*wt cells treated with CBZ was also significant (p < 0.001)

Having shown that BRG1 expression for CRC treated with CBZ is dependent on the presence of a *KRAS* mutation, we performed qPCR analysis to determine mRNA expression of *SMARCA4* after 6 and 24 h of CBZ treatment. Our qPCR analysis found results consistent with BRG1 protein expression. Results showed that in *KRAS-*mut cells, *SMARCA4* expression lowered at 24 h, resulting in a fold change of 0.69 (p < 0.01). In *KRAS-wt*, *SMARCA4* expression was raised at both 6 and 24 h after treatment, resulting in a fold change of 1.62 and 1.31 respectively (p < 0.01 and p < 0.001 respectively). The difference in fold change between *KRAS-*mut and *KRAS-wt* was statistically significant at both time points as well (p < 0.01 and p < 0.001 respectively). This supports our findings and demonstrates the opposite effect CBZ has on *KRAS-*mut vs *KRAS-wt* CRC on *SMARCA4* mRNA expression (Fig. 3).

### SMARCA4 expression may be impacted by ULK1 mRNA expression and Carbamazepine affects ULK1 expression differently in the presence of a KRAS mutation.

**Fig. 4:**
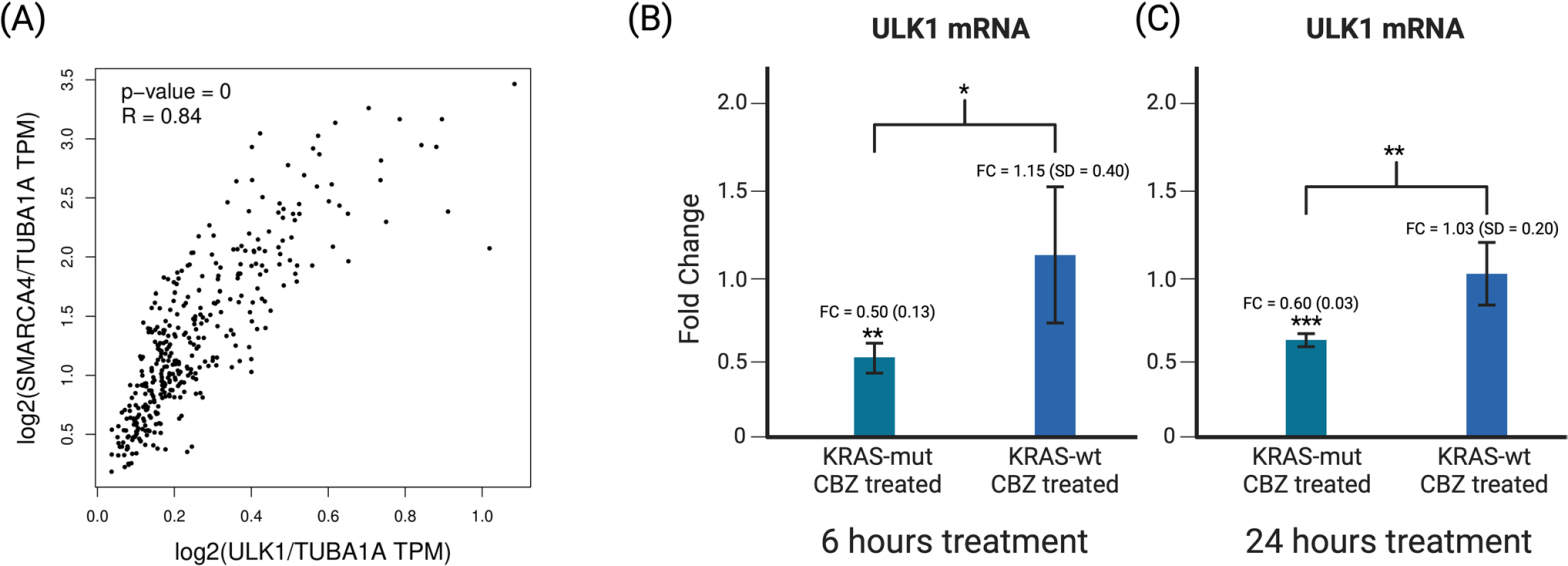
**A** Positive correlation between ULK1 and *SMARCA4* in normal tissue and colon or rectal tumors (p = 0). **B** Results are an average of 4 separate experiments. After 6 h of treatment, ULK1 mRNA levels decreased significantly in *KRAS-*mut cells treated with CBZ (p < 0.01). These levels are significantly lower than in *KRAS-*wt cells treated with CBZ (p < 0.05). **C** Results are an average of 5 separate experiments. After 24 h of treatment, ULK1 mRNA levels decreased significantly in *KRAS-*mut cells treated with CBZ (p < 0.001). These levels are significantly lower than in *KRAS-*wt cells treated with CBZ (p < 0.01)

Having shown that *SMARCA4* expression is affected by both the presence of a *KRAS* mutation as well as treatment by carbamazepine, we sought to understand the connection between these components. In patient datasets, mRNA of ULK1 and mRNA of *SMARCA4* correlated positively (p = 0, R = 0.84) (Fig. 4A). qPCR data reveals that ULK1 mRNA expression decreased in CBZ treated *KRAS-*mut CRC at both 6 and 24 h, with a fold change of 0.50 and 0.60 (p < 0.01 and p < 0.001 respectively) and was not changed in *KRAS-wt* CRC. The difference between *KRAS-*mut CRC and *KRAS-wt* CRC was significant at both time points as well. (p < 0.05 and p < 0.01 respectively) (Fig. 4B). This decrease in ULK1 corresponds to a decrease in *SMARCA4* levels and may suggest a method by which ULK1 affects *SMARCA4* in a *KRAS-*mut environment. CBZ can facilitate *KRAS-*mutated inhibition of ULK1 which may lead to a decrease in autophagy and downstream decrease in *SMARCA4* levels. In non-tumor settings, carbamazepine is known to affect ULK1 phosphorylation and thus induce autophagy [12]. Our findings suggests that CBZ may affect autophagy differently in cancers, or that the affect it has on ULK1 phosphorylation may be slightly counteracted by its effect on ULK1 mRNA levels.

### Carbamazepine preferentially binds to mutant KRAS

**Fig. 5:**
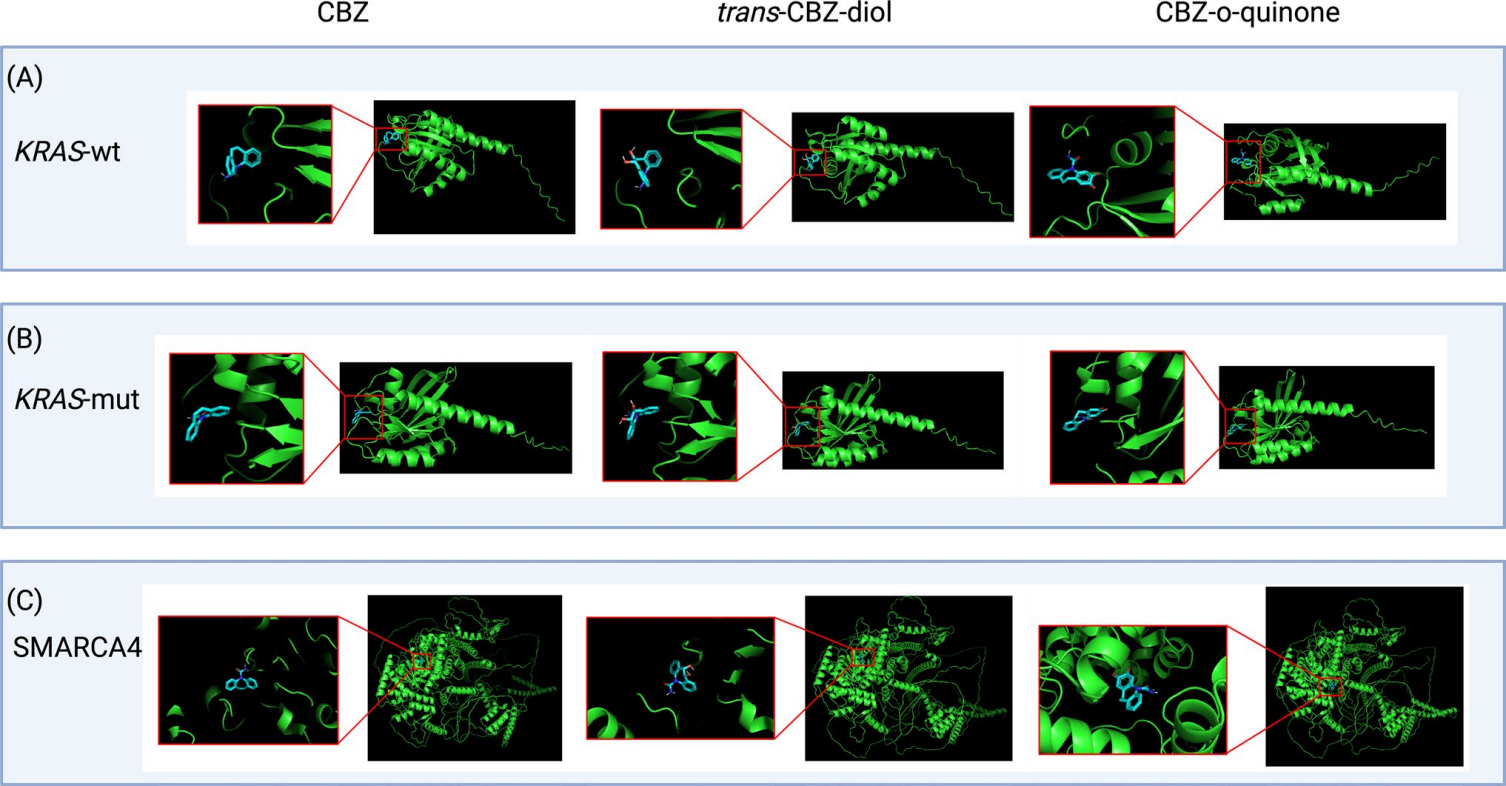
**A** WT-KRAS docked with CBZ forms shows the similar binding conformations of CBZ and t-CBZ, while CBZ-q binds in a different orientation. **B** G13D docked with CBZ forms shows similar binding conformations of CBZ and t-CBZ, while CBZ-q binds in a different orientation. The orientations of each CBZ form have visual differences than when bound to WE-KRAS. **C** *SMARCA4* docked with CBZ forms shows similar docking sites for CBZ and t-CBZ in different orientations, while CBZ-q most favorably binds elsewhere

Docking carbamazepine (CBZ) and two metabolite forms, *trans*-carbamazepine diol (*t*-CBZ) and carbamazepine-o-quinone (CBZ-*q*) with *KRAS-wt* and the G13D mutant indicated the most favorable binding form of CBZ (Table 2) and which residues participated in interaction for each (Table 3). The binding between *KRAS-wt* and G13D with each CBZ form, respectively, was visualized (Fig. 5A and B).

**Table 2:** Vina docking cores for *KRAS-*wt and G13D binding with CBZ forms

**CBZ**		***t*-CBZ**		**CBZ-*q***	
**KRAS-wt**	**G13D**	**KRAS-wt**	**G13D**	**KRAS-wt**	**G13D**
-8.2	-8.4	-7.8	-7.7	-8.5	-8.7

**Table 3:** Residues of interaction between *KRAS-*wt and G13D with CBZ forms

**CBZ**		***t*-CBZ**		**CBZ-*q***	
***KRAS*-wt**	**G13D**	***KRAS-*wt**	**G13D**	***KRAS-*wt**	**G13D**
—	Asp13	Gly13	Asp13	—	Asp13
Val14	Val14	Val14	Val14	Val14	Val14
Gly15	Gly15	Gly15	Gly15	Gly15	Gly15
Ala18	Ala18	Ala18	Ala18	Ala18	Ala18
—	—	—	—	Leu19	Leu19
Phe28	Phe28	Phe28	Phe28	Phe28	Phe28
Val29	Val29	Val29	Val29	Val29	Val29
Asp30	Asp30	Asp30	Asp30	Asp30	Asp30
Glu31	Glu31	Glu31	Glu31	Glu31	Glu31
Tyr32	Tyr32	Tyr32	Tyr32	Tyr32	Tyr32
—	—	Asp33	—	—	—
Asn85	Asn85	—	Asn85	—	—
Asn116	Asn116	Asn116	Asn116	Asn116	Asn116
Lys117	Lys117	Lys117	Lys117	Lys117	Lys117
Asp119	Asp119	Asp119	Asp119	Asp119	Asp119
Leu120	Leu120	Leu120	Leu120	Leu120	Leu120
—	—	—	—	Thr144	Thr144
Ser145	Ser145	—	—	Ser145	Ser145
Ala146	Ala146	Ala146	Ala146	Ala146	Ala146
Lys147	Lys147	Lys147	Lys147	Lys147	Lys147

The more negative that the vina docking score calculated by CB-Dock2 was, the stronger the binding through weak interactions between *KRAS-*wt and G13D and the CBZ form (Table 2). Binding energy was favored for G13D in CBZ and CBZ-*q*. However, it is notable that CBZ-*q* had the strongest binding, followed by CBZ, and lastly *t*-CBZ. There was a difference between cavity volume in *KRAS-*wt and G13D for the CBZ form binding. In *KRAS-*wt the cavity volume was 491 Å^3^, while in G13D the cavity volume was 500 Å^3^. These values were consistent with each CBZ form. Thus, the G13D mutation slightly increased the binding space.

A total of twenty residues were involved in the binding of *KRAS-*wt and G13D with the CBZ forms (Table 3). However, only fourteen of the twenty residues were consistent between the six dockings. Notably, in *KRAS-*wt the Gly13 residue only is involved in the binding with *t*-CBZ but not with CBZ or CBZ-*q*. However, when the Gly13 is mutated to Asp13, it is involved with all three forms.

Several residues appeared to bind specifically with certain CBZ forms but not others. In all instances the residues appeared in both *KRAS-*wt and G13D. The residues Leu19 and Thr144 only were involved in the binding of CBZ-*q*. The Ser145 residue was involved in the binding of CBZ and CBZ-*q* but not with *t*-CBZ. Only two residues besides Gly13 presented variability between *KRAS-*wt and G13D. Interestingly, Asn33 only participated in binding in *KRAS-*wt with *t*-CBZ. Another residue with variable results was Asn85. It participated in binding in *KRAS*-wt and G13D with CBZ, only in G13D with *t*-CBZ, and neither in *KRAS-*wt or G13D with CBZ-*q*.

CBZ, *t*-CBZ, and CBZ-*q* were also each docked with *SMARCA4* to determine which CBZ form bound best with *SMARCA4* (Supplementary Table S2) and which residues participated in interaction for each (Supplementary Table S3). The binding between *SMARCA4* with each CBZ form was visualized as well (Fig. 5C). Binding energy was similar for each CBZ form with *SMARCA4*. The cavity volume was the same between the three, measured at 8709 Å^3^.

A total of thirty-one residues were involved in the binding of *SMARCA4* with the CBZ forms (Supplementary Table S3). There were no consistent residues between the three dockings. CBZ-*q* only bound to ten residues, none of which were similar to the CBZ or *t*-CBZ dockings. CBZ and *t*-CBZ shared sixteen residues in common. Three residues, Thr910, Gly911, and Gln1185, were involved in binding with CBZ but not *t*-CBZ. Two residues, Ile187 and Glu821, were involved in binding with *t*-CBZ but not CBZ.

## Conclusion and discussion

The ATPase *SMARCA4* is overexpressed and frequently mutated in an array of cancers [10, 30]. This is supported by our analysis of CRC patient mRNA expression. We furthermore found that *SMARCA4* expression is further promoted in the presence of a *KRAS* mutation in CRC. Additionally, we have found that *SMARCA4* expression significantly correlates with patient survival exclusively for CRC patients with a *KRAS* mutation. This all suggests that *SMARCA4* inhibition serves to positively impact *KRAS-*mut patient outcomes.

Previous research on cell line SW480 (CRC *KRAS* G12V mutant) reported that CBZ may be useful in fighting cancer by decreasing β-Catenin and VEGF levels [31]. Our research supports that CBZ may also use *SMARCA4* levels as a method to affect cancer cell survival. To our knowledge, our study is the first to seek to understand the differing effect of CBZ on *KRAS-wt* and *KRAS-*mut cancer. We have found that CBZ uniquely reduces *SMARCA4* levels in *KRAS-*mutant CRC cancer alone and raises *SMARCA4* in *KRAS-wt* CRC. At both the mRNA and protein levels, *SMARCA4* is affected by CBZ. This effect was relatively consistent between time points 6 and 24 h, with the effects being more prominent at different times.

A direct correlation between *SMARCA4* and ULK1 has only been establish through *SMARCA4*’s modulation of P53 [10, 32, 33]. In this study, we found that overall *SMARCA4* correlates positively with ULK1, suggesting that P53-led ULK1 inhibition is not the primary interaction of *SMARCA4* and ULK1, and that late-stage autophagy proteins may target *SMARCA4* expression. Furthermore, while previous non cancer studies have shown that CBZ increases ULK1 [12, 13] we found that ULK1 mRNA decreased in *KRAS-*mut CRC when treated with CBZ. This suggests that CBZ may work uniquely in CRC patients, particularly those with a *KRAS* mutation. This suggests that CBZ acts differently in cancer cells when impacting autophagy and may work to support *KRAS-*induced inhibition of ULK1.

Using an in-Silico approach provides a novel examination into the unique interactions between *KRAS* and *SMARCA4*. Furthermore, it allows a blueprint to be constructed for the binding of three CBZ forms and *KRAS*, G13D, and *SMARCA4*. *KRAS*-wt interaction with *SMARCA4* had different binding patterns than the G13D mutant did. Structurally and kinetically the complexes differed in their binding mechanisms, indicating that a mutation in *KRAS* has the potential to significantly alter the interaction. CBZ had similar points of interaction with *KRAS*-wt and G13D. However, some residues varied and resulted in different binding orientations of the drug. The binding strength of the dockings favored the mutant. When docked with *SMARCA4* there was similar binding between CBZ and *t*-CBZ, although CBZ-*q* differed in a most favorable docking site.

### Supplementary Information

Below is the link to the electronic supplementary material.

Supplementary Fig. 1. **A** RMSD analysis of complex stability shows fluctuating moments of when WT-KRAS and G13D is more stable. **B** RMSF analysis of *SMARCA4* binding with WT-KRAS vs with G13D shows binding with WT-KRAS exhibits greater fluctuation around residues 300-400. **C** Rg analysis of WT-KRAS vs G13D and the *SMARCA4* in each complex shows the G13D complex is less compact. **D** Total complex energy measuring overall stability of the complexes throughout the 10ns simulations show that the WT-KRAS complex has less overall favorable binding energy but that increases, while the G13D complex has a more constant higher favorable binding energy. The more negative the energy, the more favorable binding energy. **E** Delta energy measuring the fluctuations in binding strength throughout the 10ns simulations show that the WT-KRAS complex increases in binding strength while the G13D complex fluctuates more. The more negative the energy, the more binding strength (PDF 2529 KB)

Supplementary file2 (PDF 44 KB)

Supplementary file3 (PDF 15 KB)

Supplementary file4 (PDF 48 KB)

## Data Availability

No datasets were generated or analysed during the current study.
