# Supplementary material for: Impact of carbamazepine on SMARCA4 (BRG1) expression in colorectal cancer: modulation by KRAS mutation status
Source: Invest New Drugs. 2024 Mar 6;42(2):229–39. doi: 10.1007/s10637-024-01418-2 (PMC10944448; doi:10.1007/s10637-024-01418-2)

Supplementary Figure 1

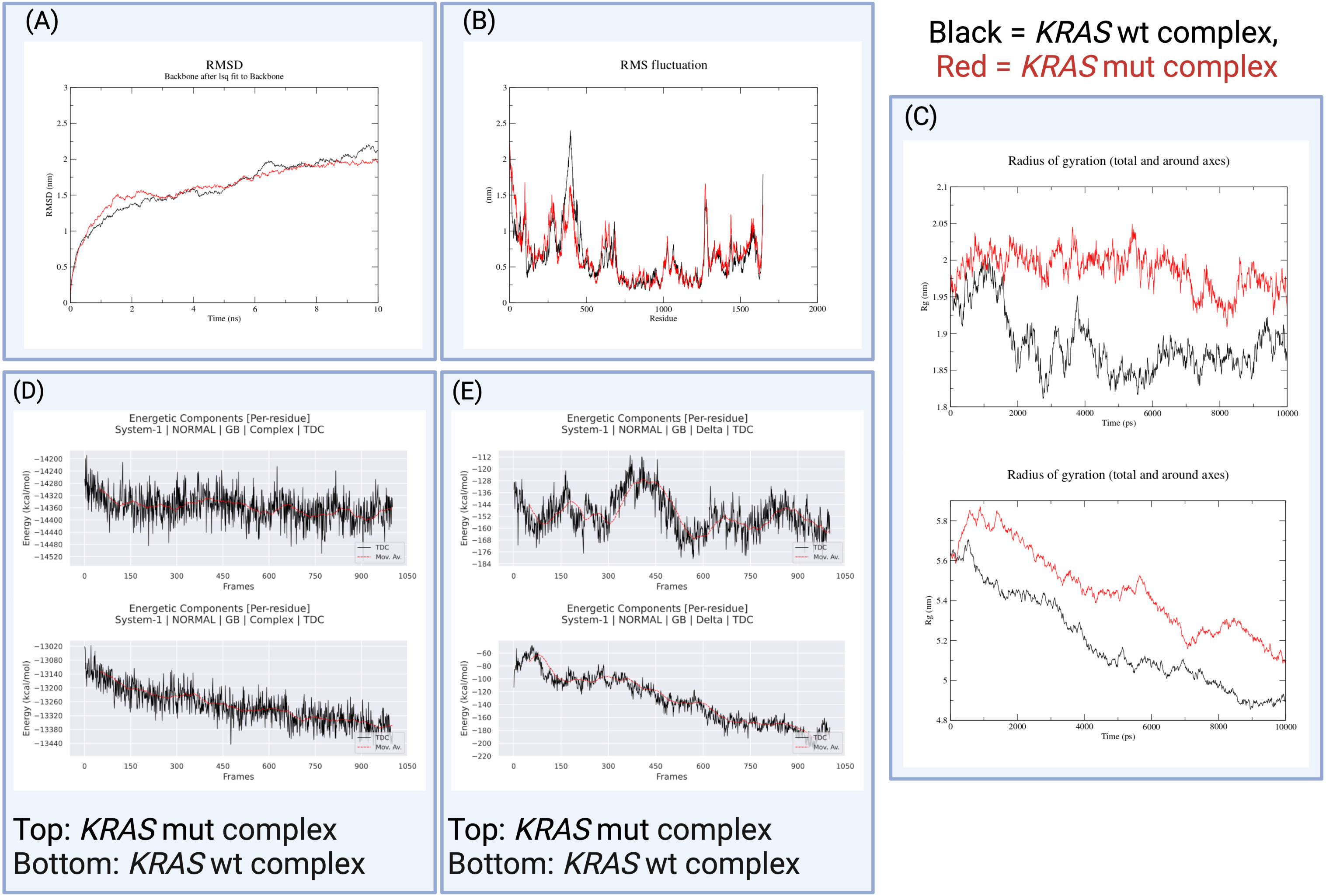

Supplement: Supplementary file 1 — Supplementary Fig. 1. A RMSD analysis of complex stability shows fluctuating moments of when WT-KRAS and G13D is more stable. B RMSF analysis of SMARCA4 binding with WT-KRAS vs with G13D shows binding with WT-KRAS exhibits greater fluctuation around residues 300-400. C Rg analysis of WT-KRAS vs G13D and the SMARCA4 in each complex shows the G13D complex is less compact. D Total complex energy measuring overall stability of the complexes throughout the 10ns simulations show that the WT-KRAS complex has less overall favorable binding energy but that increases, while the G13D complex has a more constant higher favorable binding energy. The more negative the energy, the more favorable binding energy. E Delta energy measuring the fluctuations in binding strength throughout the 10ns simulations show that the WT-KRAS complex increases in binding strength while the G13D complex fluctuates more. The more negative the energy, the more binding strength (PDF 2529 KB) [file 10637_2024_1418_MOESM1_ESM.pdf]
