# Supplementary material for: Impact of carbamazepine on SMARCA4 (BRG1) expression in colorectal cancer: modulation by KRAS mutation status
Source: Invest New Drugs. 2024 Mar 6;42(2):229–39. doi: 10.1007/s10637-024-01418-2 (PMC10944448; doi:10.1007/s10637-024-01418-2)
Supplement: Supplementary file 2 — Supplementary file2 (PDF 44 KB) [file 10637_2024_1418_MOESM2_ESM.pdf]

Supplementary Table 1. Complex and Delta energies of *KRAS*-wt and G13D Interactions with *SMARCA4*

	**Complex**	**Delta**
**KRAS-wt - *SMARCA4* Average**	-13,277.6	-133.5
**KRAS-wt - *SMARCA4* Standard Deviation**	78.3	37.6
**G13D - *SMARCA4* Average**	-14,357.7	-150.6
**G13D - *SMARCA4* Standard Deviation**	47.3	12.9
