# Supplementary material for: Impact of carbamazepine on SMARCA4 (BRG1) expression in colorectal cancer: modulation by KRAS mutation status
Source: Invest New Drugs. 2024 Mar 6;42(2):229–39. doi: 10.1007/s10637-024-01418-2 (PMC10944448; doi:10.1007/s10637-024-01418-2)
Supplement: Supplementary file 3 — Supplementary file3 (PDF 15 KB) [file 10637_2024_1418_MOESM3_ESM.pdf]

Supplementary Table 2. Vina docking cores for *SMARCA4* binding with CBZ forms

**CBZ**	***t*-CBZ**	**CBZ-*q***
-7.9	-8.0	-7.9
