# Supplementary material for: Impact of carbamazepine on SMARCA4 (BRG1) expression in colorectal cancer: modulation by KRAS mutation status
Source: Invest New Drugs. 2024 Mar 6;42(2):229–39. doi: 10.1007/s10637-024-01418-2 (PMC10944448; doi:10.1007/s10637-024-01418-2)
Supplement: Supplementary file 4 — Supplementary file4 (PDF 48 KB) [file 10637_2024_1418_MOESM4_ESM.pdf]

Supplementary Table 3. Residues of interaction between *SMARCA4* and CBZ forms

**CBZ**	***t*-CBZ**	**CBZ-*q***
Glu780	Glu780	—
Met781	Met781	—
Gly782	Gly782	—
Leu783	Leu783	—
Gly784	Gly784	—
Lys785	Lys785	—
Thr786	Thr786	—
—	Ile187	—
Thr814	Thr814	—
Asn817	Asn817	—
Trp818	Trp818	—
—	Glu821	—
Asp881	Asp881	—
Glu882	Glu882	—
Thr910	—	—
Gly911	—	—
—	—	Ile1039
—	—	Met1040
—	—	Arg1157
Gly1162	Gly1162	—
Leu1163	Leu1163	—
—	—	Asp1177
—	—	Trp1178
—	—	Asn1179
—	—	Gln1182
Gln1185	—	—
Arg1189	Arg1189	—
Arg1192	Arg1192	—
—	—	Asp1378
—	—	Tyr1879
—	—	Ser1380
